# Intergenerational Impact of Paternal Low-Protein Diet on Offspring Bone Health in Mice

**DOI:** 10.1093/function/zqaf051

**Published:** 2025-10-29

**Authors:** Slobodan Sirovica, Alexander P Morrell, Owen Addison, Richard A Martin, Adam J Watkins

**Affiliations:** Dental Physical Sciences, Barts and the London School of Medicine and Dentistry, Institute of Dentistry, Queen Mary University of London, Mile End Road, London E1 4NS, UK; Centre for Oral, Clinical & Translational Sciences, Faculty of Dentistry, Oral & Craniofacial Sciences, King’s College London, London SE1 9RT, UK; Dental Physical Sciences, Barts and the London School of Medicine and Dentistry, Institute of Dentistry, Queen Mary University of London, Mile End Road, London E1 4NS, UK; College of Engineering and Physical Sciences, Aston University, Birmingham B4 7ET, UK; School of Clinical Medicine and Population Health, University of Sheffield, Beech Hill Road, Sheffield S10 2RX, UK

**Keywords:** bone morphology, fetal programming, intergenerational effects, micro-computed tomography, paternal nutrition, X-ray diffraction

## Abstract

Our bone health as an adult is defined by patterns of development in early life, with perturbed growth during fetal and neonatal periods predisposing individuals to poor bone health in adulthood. Studies have identified poor maternal diet during pregnancy as a critical factor in shaping offspring bone development, with significant impacts on adult bone structure and health. However, the association between a father’s diet and the bone health of his offspring remains poorly defined. To address this knowledge gap, we fed male C57BL/6 mice either a control normal protein diet (NPD; 18% protein) or an isocaloric low-protein diet (LPD; 9% protein) for a minimum of 8 wk. Using these males, we generated offspring through artificial insemination, in combination with vasectomized male mating. Using this approach, we derived offspring from either NPD or LPD sperm but in the presence of NPD or LPD seminal plasma. Using micro-computed tomography and synchrotron X-ray diffraction, we observed significant changes in offspring femur morphology and hydroxyapatite crystallographic parameters from just 3 wk of age in offspring derived from LPD sperm or seminal plasma. We also observed that differential femur morphology and hydroxyapatite crystallographic parameters were maintained into adulthood and into a second generation. Analysis of paternal sperm identified a down regulation of 26 osteogenic genes associated with extracellular matrix levels and maintenance, transcription and growth factors, and bone ossification. These observations indicate that poor paternal diet at the time of conception affects offspring bone development and morphology in an age and generation specific manner.

## Graphical Abstract

**Graphical Abstract:**
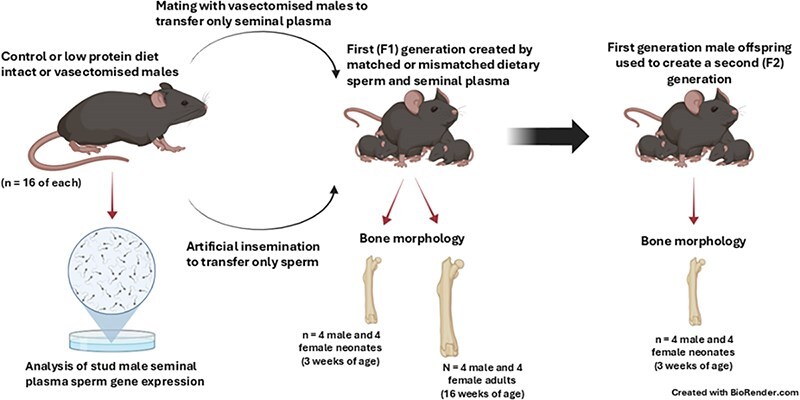

## Introduction

Our bones play a fundamental role in the provision of structural integrity for our bodies, protection of vital organs such as the brain, and the regulation of haematopoietic homeostasis. Our skeleton begins to form *in utero* via a process known as endochondral ossification, during which cartilaginous scaffolds are replaced with a matrix of mineralized bone.^1^ Our bones continue to grow throughout neonatal and early life, ceasing when the growth plates fuse, typically in our early teenage years. In contrast, bone mineral density continues to increase until our mid-to-late 20s when the process of resorption overtakes bone formation.^2^

Within the mammalian skeleton, two main types of bone exist, trabecular and cortical, with cortical bone comprising the majority of the skeletal mass. Cortical bone is characterized by a slower rate of cellular and material turnover while its greater density and extent of compaction affords a higher tolerance to bending and torsion. In contrast, trabecular bone represents a lower skeletal mass but has a significantly higher bone area and volume than cortical bone.^3^ Trabecular bone, which has a higher degree to turnover than cortical bone, is found inside the long and flat bones of the body as well as the vertebrae, where it acts to provide mechanical support.^4^

Throughout our lives, bones undergo continual phases of remodeling, consisting of osteoclast-directed digestion and resorption of old bone and osteoblast-directed creation and deposition of new bone.^2^ This remodeling serves to adapt the bone to the changing mechanical forces applied upon it during the lifetime of an individual. Underlying the remodeling of bone is a range of local and systemic factors including hormones, such as parathyroid hormone, glucocorticoids, growth hormone, calcitriol, and the sex hormones.^5^ Therefore, maintaining appropriate bone health is often viewed as a continual balance between the resorption of old, damaged bone and the deposition of new bone.

Bone health in adulthood is viewed typically as a product of our ongoing lifestyle and physiological factors. However, a significant body of data has highlighted the importance of our development prior to birth. Studies have shown that poor maternal diet during gestation can impair fetal skeletal development and adult offspring bone health.^6^ In mice, maternal high-fat diet during gestation delays skeletal formation, resulting in lower bone mineral density, bone volume, and bone formation.^7,8^ Maternal undernutrition, in response to a low-protein diet, has also been shown to influence fetal bone developmental dynamics, with bigger effects in male offspring than in females.^9^ In adult mice, the bones of offspring of dams fed a high-fat diet also display reduced bone mineral density and bone volume, especially of the long bones.^10,11^ Similar to the influences of poor maternal diet, the role of the father’s nutrition at the time of conception for regulating offspring development is now an area of active research.^12^ In mice, males fed a diet low in folate sired offspring displaying increased incidence of birth defects including craniofacial and musculoskeletal malformations.^13^ Similarly, our own studies have shown that fetal growth and bone development are altered in response to a paternal low-protein diet in mice.^14^ Underlying the paternal programming of offspring development are significant changes in sperm epigenetic status.^13,15,16^ Furthermore, studies have identified a significant role of the seminal plasma in regulating the maternal uterine environment and the developmental trajectory of the offspring.^16-18^ These seminal plasma-induced responses are essential for preparing the uterine tissue ahead of embryo implantation and for modulating the maternal immune system toward paternal antigens. Previously, we have shown that offspring metabolic and cardiovascular health are impaired via specific sperm and seminal plasma-mediated mechanisms over multiple generations.^19,20^

While a picture of the impact of poor paternal fitness on the cardio-metabolic health of his offspring is emerging, the consequences for the structure and composition of the bones remains undefined. In the current study, we have used our existing mouse model to define the impact of paternal diet on offspring bone development and structure. Furthermore, we define the specific sperm and seminal plasma roles in regulating offspring bone formation and structure and study their impact over multiple generations.

## Materials and Methods

### Animal Dietary Manipulation

All animal experimental procedures were conducted under the UK Home Office Animal (Scientific Procedures) Act 1986 Amendment Regulations 2012, which transposed Directive 2010/63/EU into UK law, with approval of the local ethics committee at Aston University. Intact and vasectomized 8-wk old C57BL/6 male mice (Harlan Ltd, Belton, Leicestershire, UK) were maintained as described previously.^16,20^ Briefly, males were fed either control normal protein diet (NPD; 18% casein; *n* = 16 intact and 8 vasectomized males) or isocaloric low-protein diet (LPD; 9% casein; *n* = 16 intact and 8 vasectomized) for a period of 8-12 wk to ensure all stages of spermatogenesis and spermiogenesis were exposed to the diets.^21^ Diets were manufactured commercially (Special Dietary Services Ltd; UK) and their composition is provided in Supplemental Table S1. Virgin 8-wk old female C57BL/6 mice (Charles River, UK), were maintained at Aston University’s biomedical research facility on a 07:00-9:00 light-dark cycle at a temperature of 20-22°C with *ad libitum* access to chow and water.

### Generation of F1 and F2 Offspring

Offspring were generated as described previously.^16^ Virgin females were superovulated with 1IU pregnant mare serum gonadotrophin, followed 48 h later with 1IU human chorionic gonadotrophin (Intervet, UK). Twelve hours after human chorionic gonadotrophin injection, stud NPD and LPD fed males were culled by cervical dislocation for the collection of mature, motile epididymal sperm. Sperm were allowed to swim out and capacitate for 90 min at 37°C (5% CO_2_ in air) in swim out medium (135 m m NaCl, 5 m m KCl, 1 m m MgSO4, 2 m m CaCl2, 30 m m HEPES; supplemented freshly with 10 m m lactic acid, 1 m m sodium pyruvate, 20 mg/mL BSA, 25 m m NaHCO_3_). Females were non-surgically artificially inseminated with 50 µL of sperm (approximately 10^7^ sperm) in swim out medium. Immediately following artificial insemination, females were placed overnight with an NPD or LPD fed vasectomized male. The presence of a vaginal plug the following morning indicated successful mating. All females received standard chow and water *ad libitum* and were allowed to develop to term with their pregnancies. This experimental insemination and mating strategy generated 4 groups of offspring termed “NN” (NPD sperm and NPD seminal plasma), “LL” (LPD sperm and LPD seminal plasma), “NL” (NPD sperm and LPD seminal plasma), and “LN” (LPD sperm and NPD seminal plasma). All offspring received standard chow and water *ad libitum* throughout the study. For the production of an F2 generation, 16-wk-old F1 males (*n* = 6 males per treatment group; each from separate litters) were mated naturally to virgin, 8-wk old female C57BL/6 mice (Charles River, UK) which were acquired separately for the purpose of mating with F1 males. All F2 offspring received standard chow and water *ad libitum*. All animals (sires, dams, and offspring) were culled via cervical dislocation.

### Stud Male Seminal Plasma Analysis

Seminal plasma was collected from both seminal vesicles of intact NPD and LPD stud males into 200 µL of sterile PBS with 0.5% BSA (Sigma Aldrich, UK), weighed, mixed, centrifuged at 7000 x g for 3 min and the supernatant removed. Total protein levels in the supernatant were determined using the Qubit fluorometer (ThermoFisher Scientific, UK) and the Qubit protein assay kit (Molecular Probes Technologies, UK), in accordance with the manufacturer’s instructions, prior to being stored at −80°C. Levels of adiponectin, leptin, and Tgfb1 were determined in seminal plasma supernatants using commercial ELISAs (catalogue #s MRP300, MOB00, and DY1679, respectively; R&D systems) in accordance with the manufacturer’s instructions. Stud males were typically 20 wk of age at the time of cull.

### Stud Male Sperm Transcript Expression

Mature sperm were retrieved from the caudal epididymis of NPD and LPD stud males following cull. Both epididymides were roughly sliced in warmed M2 media (M7167- Sigma Aldrich, UK) and were left for 30 min at 37°C for the sperm to swim up into fresh media. The total live sperm fraction was collected and snap frozen. Sperm total RNA was extracted using Qiagen miRNeasy micro kit following manufacturer’s instructions with homogenization in Qiazol using TissueLyser II. Additional on-column DNase I digestion (Qiagen, UK), was conducted prior to cDNA synthesis (nanoScript 2 reverse transcription kit; Primerdesign, UK), all according to manufacturer’s instructions. Expression of 84 Osteogenesis pathway genes were analyzed using a PCR array (GeneGlobe ID—PAMM-026Z; Qiagen, UK) in accordance with the manufacturer’s instructions. Amplification was performed on a Stratagene Mx 3000P System (Agilent Technologies, USA) with resultant gene expression analyzed using web-based PCR Array Data Analysis software (www.SAbiosciences.com).

### Offspring Femur Micro-Computed Tomography Analysis

F1 male and female offspring were culled at either 3 (neonatal) or 16 (adult) weeks of age while all F2 offspring were culled at 3 wk of age. Offspring femurs were dissected free from muscle prior to fixation in 4% neutral buffered formalin (Sigma Aldrich, UK) at 4°C overnight (16 h) and subsequent storage in 70% ethanol prior to analysis. An additional set of adult F1 offspring femurs were embedded into epoxy resin and sectioned at a thickness of approximately 1 mm for the production of thin, transverse sections of cortical bone, using the third trochanter as an anatomical reference point for each bone.

Whole, fixed femurs from male and female F1 neonatal and adults, and F2 neonatal NN, LL, NL, and LN offspring were scanned using a Skyscan 1174 micro-computed tomography (µ-CT) scanner (Bruker, Belgium). All scans were taken at 50 kVa and 800 µA with a 0.5 mm aluminium filter, 3600 ms exposure time, 180° tomographic rotation and a voxel resolution of 17.84 µm^2^. Individual two-dimensional cross-sectional images were reconstructed using Bruker NRecon software (version 1.7.4.6). Stacks of images for each individual femur were imported into BoneJ (version 7.0.14)^22^ and the total number of sections was defined. Identification of the trabecular and cortical regions for analysis was defined based on the first appearance of a bridging connection of low-density growth plate chondrocyte seam. Using this as a standard, established anatomical set point, an offset of 3% of the total bone length toward the femoral head from the reference growth plate was used to define the start of the trabecular region of interest, and a series of trabecular sections representing 5% of the total bone length was analyzed. For the analysis of the cortical bone, the mid bone position was defined based on the total length of the bone and an offset of 2.5% of the total bone length toward the femoral head was used to define the start of the cortical region. A series of cortical sections representing 5% of the total bone length was analyzed. Using BoneJ, measurements of trabecular and cortical bone volume (Bv), total volume (Tv), bone volume fraction (Bv/Tv), trabecular thickness (Tb.Th) and spacing (Tb.Sp), medullary cavity volume (Mv) degree of anisotropy (Da), connectivity density (Conn.D), maximum moment of inertia (Imax), and minimum moment of inertia (Imin) were defined. In addition, cortical bone cross section area (Cb-Cx) and thickness (Bc-Th) and the volume of the medullary cavity (Mv) were also defined.

### Synchrotron X-Ray Diffraction

Synchrotron X-ray diffraction (SXRD) measurements of F1 and F2, male and female NN, LL, NL, and LN offspring whole femurs and cortical ring sections were conducted at beamlines B16, I16, and I18 at Diamond Light Source (Oxford Harwell Campus, Didcot, UK) and the XMaS (BM28) beam line at the European Synchrotron Radiation Facility. Whole femurs and cortical ring sections were mounted normal to the impinging X-rays in transmission geometry and on a X-Y translational stage to allow measurements in 2 orthogonal directions perpendicular to the X-ray beam. Dependent on the beam line used, incident X-ray energies between 14.5 and 17 KeV were used, equivalent to a wavelength (λ) range of 0.7293-0.855 Å, with a beam size range of 10-35 µm (horizontal and vertical). For whole femurs, single line transects were taken immediately below the third trochanter, scanned in 10-25 µm increments and an exposure time of 30-60 s. For ring sections of cortical bone, 2 orthogonal line transects were taken, scanning across the entire face of the bone section (including meduallary cavity). Diffraction data were collected using 2D area detectors (Image Star 9000, pixel size = 31 × 31 µm; Photonic Science Ltd UK on B16; Pilatus 100 K detector, 100 K × 172 × 172 µm pixel photon counting detector on I16; Pilatus P3-2 M, Dectris, Baden-Daettwil, Switzerland, 475 × 1679 pixel array format—pixel size = 172 × 172 µm on I18 and a MAR165 CCD detector, 2048 × 2048 pixels, pixel size = 80 × 80 µm^2^, on XMaS) each placed between 100-200 mm behind the sample to give a 2θ range of 4-55°, corresponding to a q range of 0.53-7.02 Å^−1^. All measurements and instrument parameters were determined against silicon, alumina, and/or lanthanum hexaboride (LaB_6_) standards. In addition, measurements were taken also for the direct beam, empty sample containers, beryllium windows, and water.

### SXRD and Data Analysis

To capture intensity variations in diffraction rings ie, texture effects, images were sectioned into 5° slices (72 slices per diffraction image), with each slice azimuthally integrated to produce 1D data of intensity (*I*) versus the scattering angle (2θ) for Rietveld refinement.^23^ Rietveld refinement was used to determine changes in the crystallographic-lattice parameters using GSAS-II software (General Structure Analysis System-II, version 5046, 2022, Argonne National Laboratory, Illinois, USA).^24^ A hexagonal unit cell with a P63/m symmetry space group was assumed for the hydroxyapatite structural model with initial lattice parameters adopted from Young.^25^ Instrument parameters including X-ray wavelength, sample to detector distance, and peak-shape profile were determined using a LaB_6_ calibrant standard sample. These parameters were kept fixed for the refinement of the data. The scale and background parameters (a 9 term Chebyshev function and three pseudo-Voigt peak shape profiles modeling the signal contributions arising from amorphous components) were initially refined. Lattice parameters [unit-cell axes (*a* = *b* and *c*)], sample displacement and domain size were refined next, followed by texture parameters modeled using a spherical harmonics function. The quality of the refinement was determined by least squares methods where the goodness of fit increased as *χ*^2^ approached unity.

### Statistical Analyses

Data were assessed for normality (Shapiro-Wilk test) using SPSS (Version 24). Analysis of bone morphology was conducted using a multilevel random effects regression model (SPSS version 24, 2018), adjusting paternal origin of litter, gestational litter size, and body weight effects where appropriate. Where a significant effect of sex was observed, data for each sex were analyzed separately and reported as such. Significance was taken at *P* < 0.05.

## Results

### F1 Neonatal Offspring

**Figure 1.**
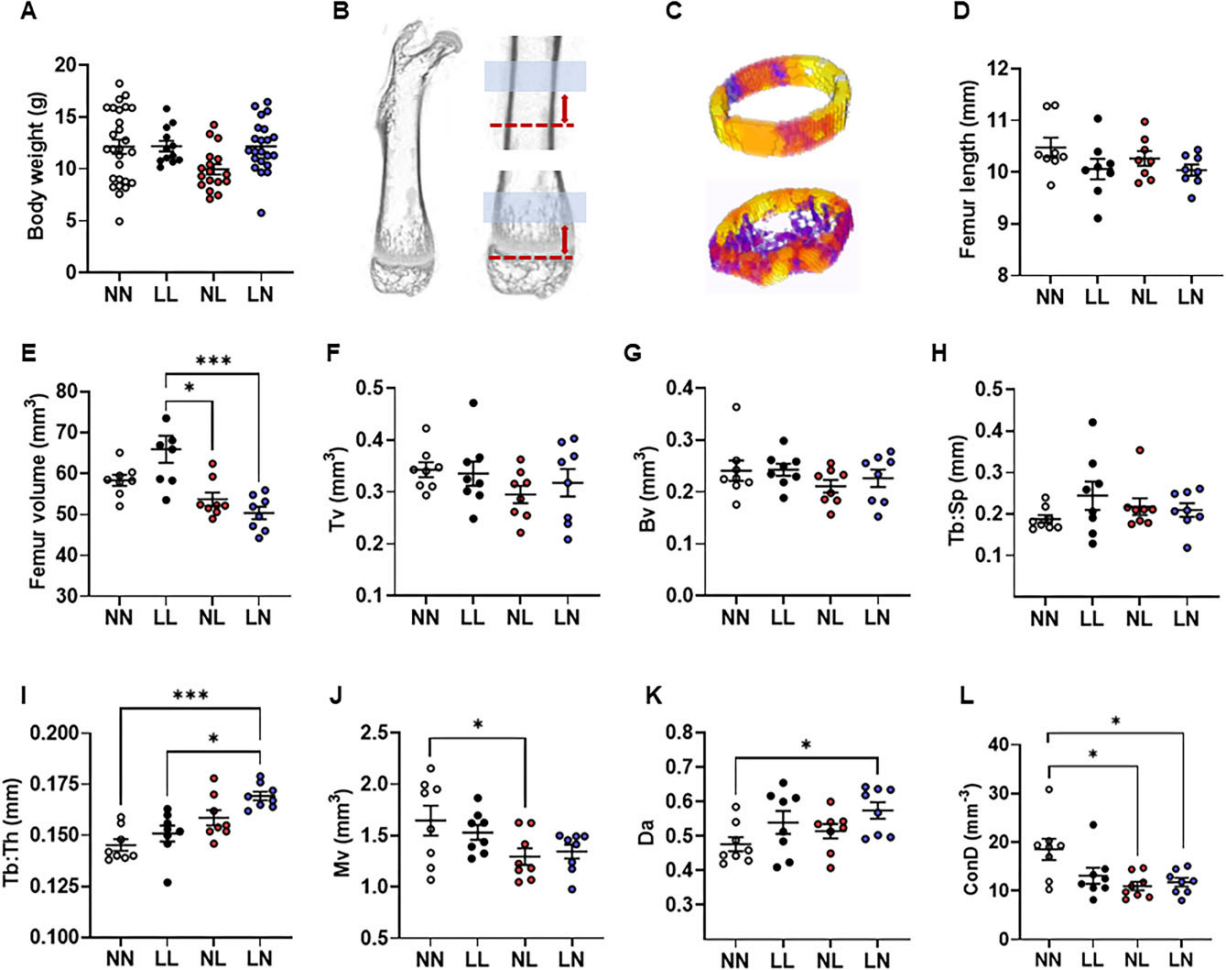
Impact of paternal diet on F1 neonatal offspring femur morphology. (A) Body weight at 3 wk of age in NN (NPD sperm and NPD seminal plasma), LL (LPD sperm and LPD seminal plasma), NL (NPD sperm and LPD seminal plasma) and LN (LPD sperm and NPD seminal plasma) offspring. (B) Representative longitudinal µ-CT scan of a whole neonate femur highlighting the anatomical regions selected for the analysis of (C) trabecular and cortical bone. (D) Whole femur length and (E) volume in NN, LL, NL, and LN offspring. (F) Femur trabecular total volume (Tv), (G) bone volume (Bv), (H) trabecular spacing (Tb: Sp) and (I) thickness (Tb: Th), (J) medullary volume (Mv), (K) degree of anisotropy (Da) and (L) connectivity density (Con.D) in NN, LL, NL, and LN offspring. *N* = 12-27 offspring in A and 8 offspring (4 males and females, B-L) per treatment group, sampled from all litters generated. Data are expressed as mean ± SEM. ^∗^*P* < 0.05, *** *P* < 0.001. Statistical differences were determined using a one-way ANOVA with Bonferoni post-hoc correction (A) or Kruskal-Wallis with Dunn’s multiple comparison test (B-L).

Details of first generation NN, LL, NL, and LN litter size, growth, organ sizing, cardiovascular, and metabolic health for up to 16 wk of age have been published previously.^16,19,20^ At 3 wk of age, there was no effect of diet (*P* = 0.285) or sex (*P* = 0.887) on offspring body weight (Figure 1A). As there was no significant effect of sex (*P* = 0.562), data for males and females were combined. We analyzed offspring femur bone morphology using µ-CT, focusing specifically on the regions of trabecular and cortical bone (Figure 1B, C). While no difference in femur length was observed among groups (Figure 1D), NL and LN offspring displayed significantly decreased whole femur volumes when compared with LL offspring (Figure 1E; *P* < 0.05). In the trabecular bone, no difference in total bone volume (Tv; Figure 1F) or trabecular bone volume (Bv; Figure 1G) were observed among groups. While there were no differences in trabecular separation between groups (Tb: Sp; Figure 1H), NL and LN offspring displayed a significantly increased trabecular thickness when compared with NN and LL offspring (Tb: Th; Figure 1I; *P* ≤ 0.05). We also observed that medullary cavity volume was significantly decreased in NL offspring when compared with NN offspring (Mv; Figure 1J; *P* = 0.05). Finally, while LN offspring displayed an increased degree of anisotropy in the trabecular bone when compared with NN offspring (Da; Figure 1K; *P* = 0.033), NL and LN offspring displayed a reduced connectivity density when compared with NN offspring (ConD; Figure 1L; *P* < 0.05).

**Figure 2.**
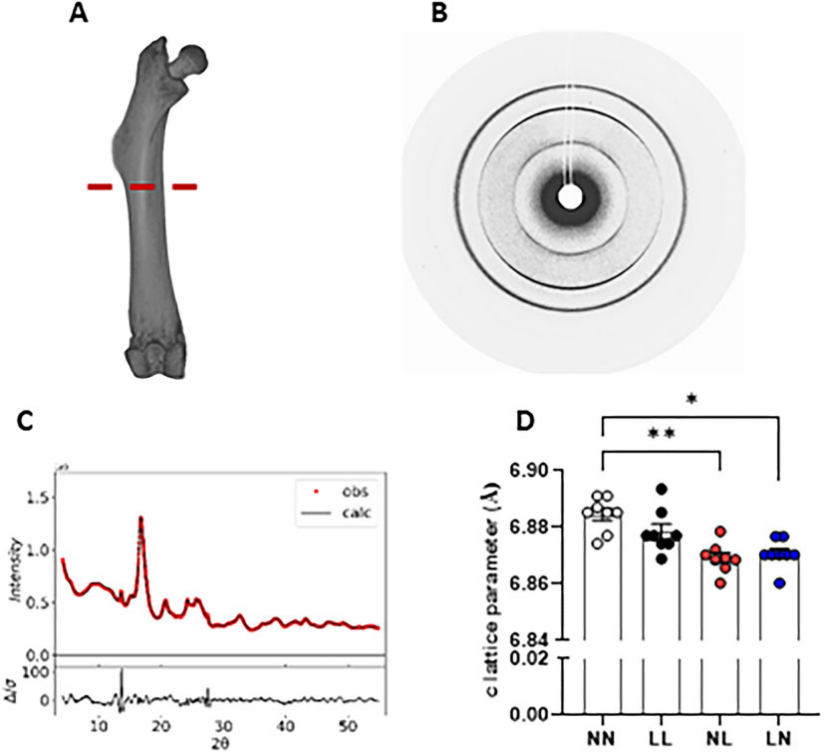
Impact of paternal diet on F1 neonatal offspring femur hydroxyapatite lattice parameters. (A) Representative image of a neonatal (3 wk old) femur indicating (dotted line) the region immediately under the third trochanter where SXRD scans were taken. (B) Representative example of an X-ray diffraction pattern showing characteristic 002 and 300 rings and (C) the full pattern fitting of the data. (D) Femur hydroxyapatite c-lattice parameter in NN (NPD sperm and NPD seminal plasma), LL (LPD sperm and LPD seminal plasma), NL (NPD sperm and LPD seminal plasma) and LN (LPD sperm and NPD seminal plasma) offspring. *N* = 8 offspring (4 males and females) per treatment group, sampled from all litters generated. Data are expressed as mean ± SEM. ^∗^*P* < 0.05, **P < 0.01. Statistical differences were determined using a one-way ANOVA with Bonferoni post-hoc correction.

Next, we conducted analysis of F1 neonatal offspring femur hydroxyapatite lattice parameters using SXRD. Scanning laterally across the bone just under the third trochanter (Figure 2A), we obtained diffraction data on reflection (Figure 2B) which were refined and fitted to give measurement of c-lattice (13° scattering angle) parameters (Figure 2C). We observed that NL and LN offspring displayed a reduced c-lattice parameter when compared with NN offspring (Figure 2D; *P* < 0.05).

### F1 Adult Offspring

**Figure 3.**
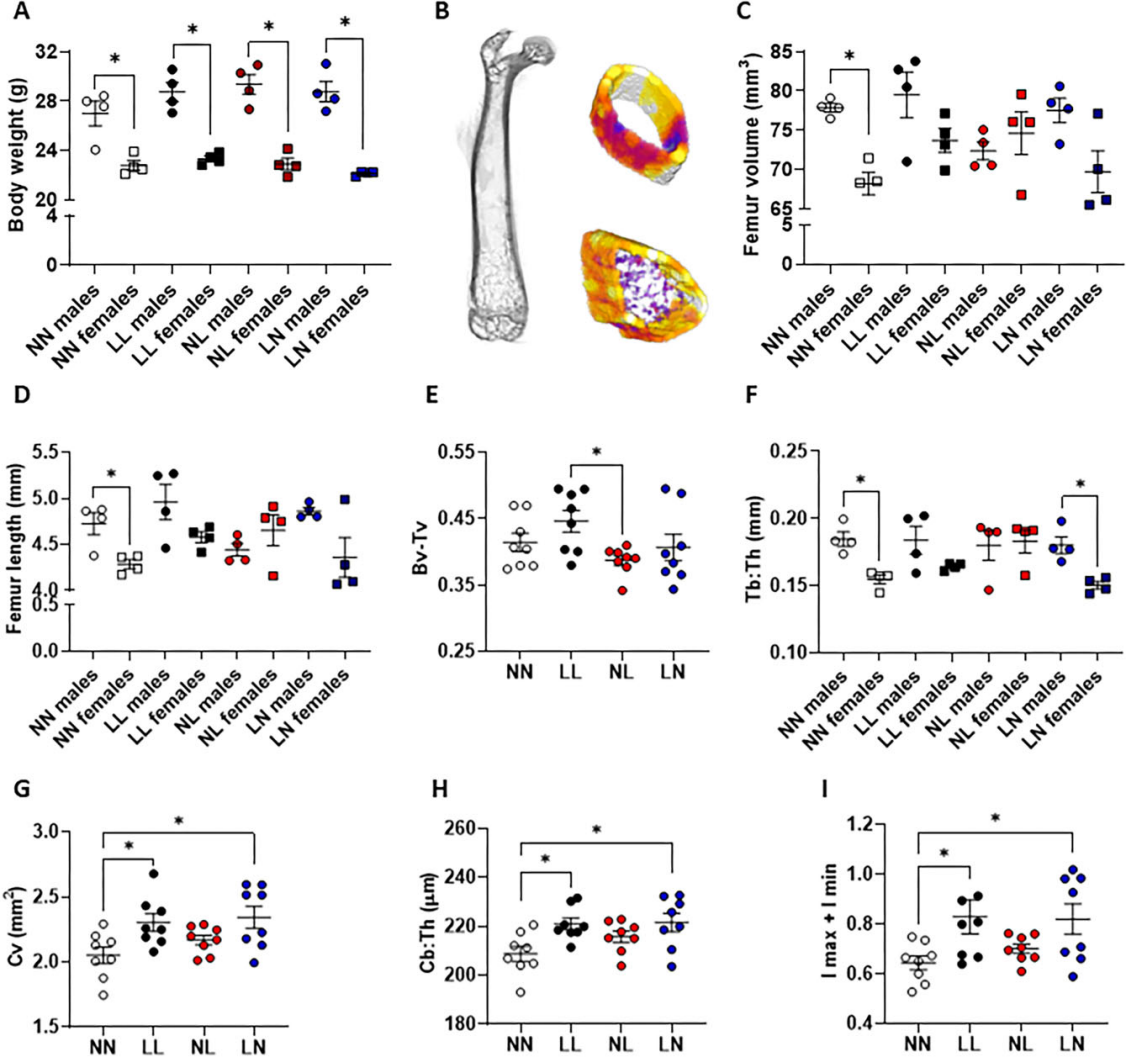
Impact of paternal diet on F1 adult offspring femur morphology. (A) Body weight at 16 wk of age in NN (NPD sperm and NPD seminal plasma), LL (LPD sperm and LPD seminal plasma), NL (NPD sperm and LPD seminal plasma), and LN (LPD sperm and NPD seminal plasma) males and females. (B) Representative longitudinal µ-CT scan of a whole neonate femur and examples of the trabecular and cortical bone regions analyzed. (C) Whole femur volume and (D) length in NN, LL, NL, and LN males and females. (E) Trabecular bone volume fraction (Bv-Tv), (F) trabecular thickness (Tb-Th) in males and females, (G) cortical bone volume (Cv), (H) cortical bone thickness (Cb-Th) and (I) cortical bone robustness (sum of *Imax* and *Imin*). N = 8 offspring (4 males and females) per treatment group, sampled from all litters generated. Data are expressed as mean ± SEM. ^∗^*P* < 0.05. Statistical differences were determined using a one-way ANOVA with Bonferoni post-hoc correction or Kruskal-Wallis with Dunn’s multiple comparison test.

In our adult offspring, we observed that in all groups, males were significantly heavier than their respective females at 16 wk of age (Figure 3A; *P* < 0.03). However, there were no effects of diet on the weight of males (*P* = 0.49), or females (*P* = 0.10) on the animals analyzed. Despite these sex-specific differences in body weight, analysis of F1 adult trabecular and cortical bone morphology (Figure 3B) showed that only NN offspring displayed a sex-specific difference in femur volume (Figure 3C; *P* = 0.03) and length (Figure 3D; *P* = 0.03). In the trabecular bone, NL offspring displayed a lower ratio of bone volume: total bone volume when compared with LL offspring (Bv: Tv; Figure 3E; *P* = 0.046). Furthermore, while there were no differences in mean trabecular thickness between the groups (effect of diet *P* = 0.055), we did observe that NN and LN offspring displayed significant sex-specific differences (Figure 3F) with females having a lower mean thickness than their respective males (*P* < 0.03). In the cortical bone, we observed a larger proportional volume of cortical bone in LL and LN offspring when compared with NN offspring (Cv; Figure 3G; *P* < 0.05). We also observed an increase in the thickness of the cortical bone (Cb: th; Figure 3H; *P* < 0.05) and an increase in cortical bone robustness (sum of Imax and Imin) (Figure 3I; *P* < 0.05) in LL and LN offspring when compared with NN offspring bones.

**Figure 4.**
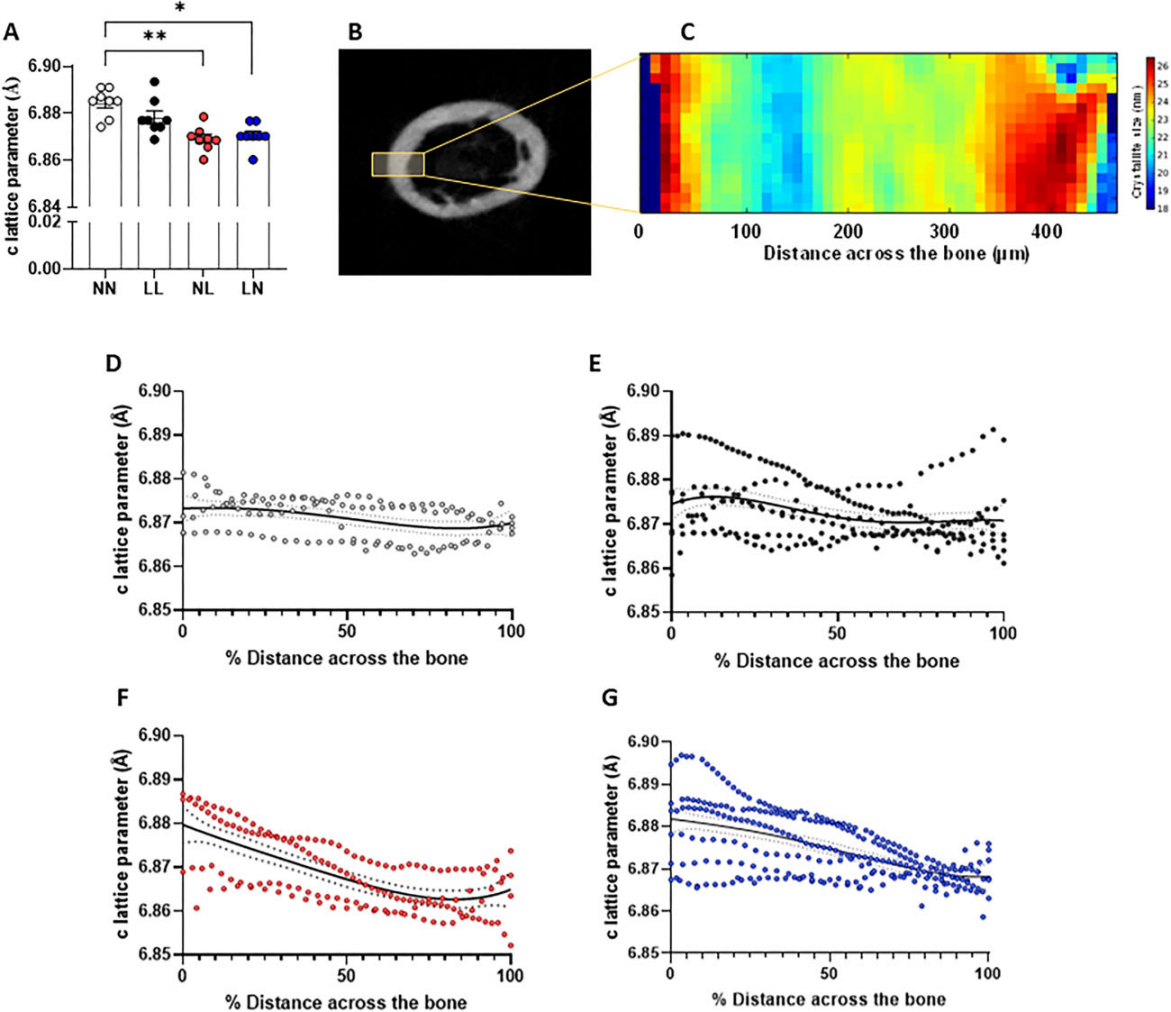
Impact of paternal diet on F1 adult offspring femur hydroxyapatite lattice parameters. (A) Average adult (16 wk old) femur cortical ring section hydroxyapatite c-lattice parameter in NN (NPD sperm and NPD seminal plasma), LL (LPD sperm and LPD seminal plasma), NL (NPD sperm and LPD seminal plasma) and LN (LPD sperm and NPD seminal plasma) adult offspring. (B) Representative femur cortical section indicating the region analyzed for SXRD mapping with (C) a representative map of crystallite size across the bone. Analysis of c-lattice parameter across (D) NN, (E) LL, (F), NL, and (G) LN cortical ring sections, moving from the outer periosteum (0% distance across the bone) to the medullary cavity (100% distance) across the bone. *N* = 4 offspring (2 males and 2 females) per treatment group, sampled from all litters generated. Data are expressed as mean ± SEM in A, B, and C. ^∗^*P* < 0.05. Statistical differences were determined using a one-way ANOVA with Bonferoni post-hoc correction or Kruskal-Wallis with Dunn’s multiple comparison test.

Analysis of femur hydroxyapatite parameters from line transects taken across the outer face of the bone and under the third trochanter, revealed no effect of sex (*P* = 0.823) on mean c-lattice parameter. However, both NL and LN bones displayed a reduced c-lattice parameter (Figure 4A) when compared with NN offspring (*P* < 0.05). Next, we undertook cross sectional mapping of thin cortical bone ring sections transecting across the entire face of the bone and medullary cavity (Figure 4B, C). In NN bones, we observed a slight decrease in c-lattice parameter as we scanned from the outer periosteum toward the medullary cavity (deemed 100% distance across the bone) (Figure 4D). While bones from LL offspring displayed the same slight decrease in c-lattice parameter, their bones showed much a higher variability, with some bones even displaying an increase toward the medullary cavity (Figure 4E). In NL and LN bones, we observed a more pronounced decrease in c-lattice parameter as we progressed toward the medullary cavity (Figure 4F, G), with high inter-bone variability in the LN bones (Figure 4G).

### F2 Neonatal Offspring

**Figure 5.**
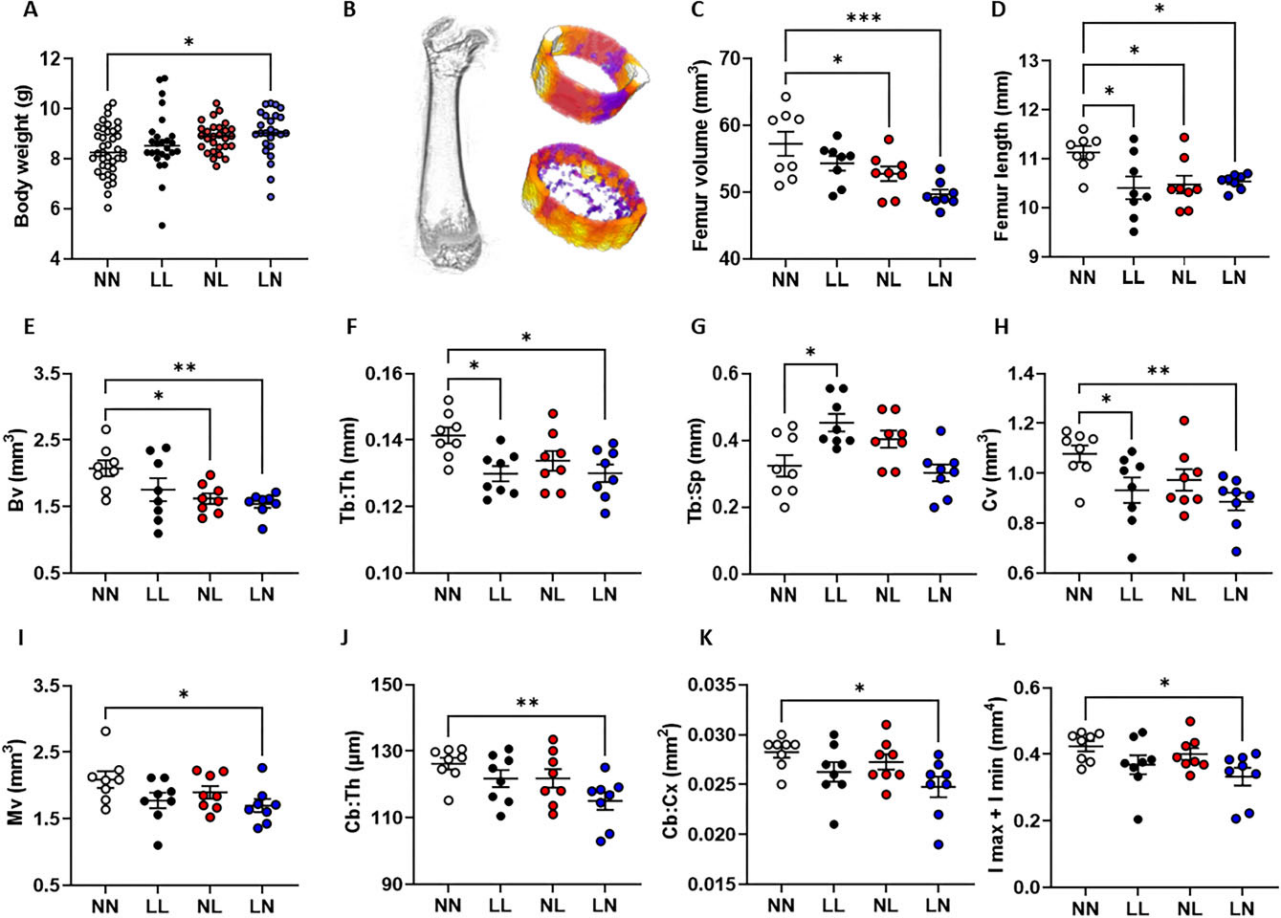
Impact of paternal diet on F2 neonatal offspring femur morphology. (A) Body weight at 3 wk of age in NN (NPD sperm and NPD seminal plasma), LL (LPD sperm and LPD seminal plasma), NL (NPD sperm and LPD seminal plasma), and LN (LPD sperm and NPD seminal plasma) F2 offspring. (B) Representative longitudinal µ-CT scan of a whole neonate femur and examples of the trabecular and cortical bone regions analyzed. (C) Whole femur volume and (D) length in NN, LL, NL, and LN F2 offspring. (E) Femur trabecular bone volume (Bv), (F) trabecular thickness (Tb: Th), (G) trabecular spacing (Tb: Sp), (H) cortical bone volume (Cv), (I) cortical medullary volume (Mv), (J) cortical bone thickness (Cb: Th), (K) cortical bone cross sectional area (Cb: Cx), and (L) cortical bone robustness (sum of *Imax* and *Imin*) in NN, LL, NL, and LN offspring. *N* = 8 offspring (4 males and females) per treatment group, sampled from all litters generated. Data are expressed as mean ± SEM. ^∗^*P* < 0.05. Statistical differences were determined using a one-way ANOVA with Bonferoni post-hoc correction or Kruskal-Wallis with Dunn’s multiple comparison test.

To determine whether changes in offspring bone structure and morphology were maintained across generations, we created an F2 generation using our adult F1 males. Details on F2, 3 wk old neonatal offspring growth, cardiovascular and metabolic profiles have been reported previously.^19,20^ As we observed no differences in F2 neonatal bone morphology between male and female offspring (*P* = 0.35), data for males and females were combined. At 3 wk of age, LN offspring were significantly heavier than NN offspring (Figure 5A; *P* = 0.04). Analysis of F2 neonatal offspring femur trabecular and cortical bone morphology (Figure 5B) revealed a significantly decreased whole bone volume in NL and LN offspring when compared with NN offspring (Figure 5C; *P* < 0.05), while femur length was decreased in LL, NL, and LN and bones (Figure 5D; *P* < 0.05). In the trabecular bone, we observed a reduced relative trabecular bone volume in NL and LN bones (Bv; Figure 5E; *P* < 0.03). Trabecular thickness was decreased significantly in LL and LN bones when compared with NN bones (Tb: Th; Figure 5F; *P* < 0.035), while trabecular separation was increase in LL bones when compared with NN bones (Tb: Sp; Figure 5G; *P* = 0.05). In the cortical bone, LL and LN bones displayed a decreased cortical bone volume (Cv; Figure 5H; *P* < 0.05), while LN bones also displayed a reduced medullary cavity volume (Mv; Figure 5I; *P* = 0.045), reduced cortical bone thickness (Cb: Th; Figure 5J; *P* = 0.01) and cortical bone cross section area (Cb: Cx; Figure 5K; *P* = 0.02), Finally, analysis of a femur cortical geometry and bone robustness (the sum of *Imax* and *Imin*), revealed LN bones had a decreased percentage of cortical area with respect to the area of the entire cross-section when compared with NN bones (Figure 5L; *P* = 0.04). Analysis of F2 neonate femur hydroxyapatite c-lattice parameters revealed no differences in the c-lattice parameter between groups (See Supplemental Figure S1).

**Figure 6.**
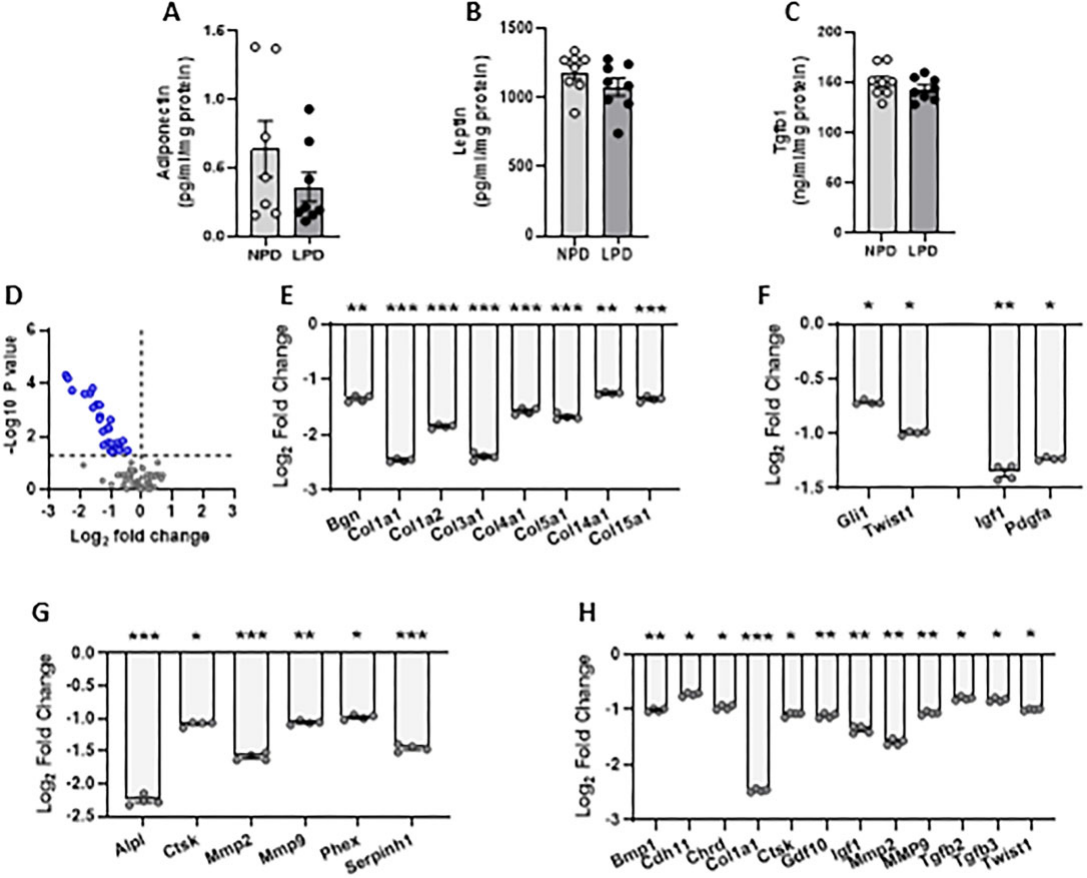
Analysis of NPD and LPD stud male seminal plasma and sperm composition. (A) Seminal plasma levels of adiponectin, (B) leptin and (C) Tgfb1 in NPD and LPD fed stud males. (D) Sperm expression of osteogenesis genes using a qPCR array in NPD and LPD fed stud males. Mean relative expression of sperm genes associated with (E) extracellular matrix, (F) transcription factors and growth factors, (G) ECM protease function, and (H) bone ossification. *N* = 7-8 males per group. Data are expressed as mean ± SEM in A-C. Statistical differences were determined using a one-way ANOVA with Bonferoni post-hoc correction or Kruskal-Wallis with Dunn’s multiple comparison test in A-C and the web-based PCR Array Data Analysis software (www.SAbiosciences.com) in E-F.

Our data indicate that inter-generational offspring bone development and structure are perturbed in response to both sperm- and seminal plasma-specific mechanisms, mirroring our previous analysis of offspring cardiovascular^20^ and metabolic^19^ health. To gain additional insight into the mechanism through which poor paternal diet might program offspring bone development, we analyzed seminal plasma composition and sperm mRNA profiles in our F0 stud males at approximately 20 wk of age. We observed no difference in the levels of seminal plasma adiponectin (Figure 6A), leptin (Figure 6B), or Tgfb1 (Figure 6C) between our NPD and LPD fed males. Next, we conducted a quantitative PCR array for 86 osteogenesis genes (see Supplemental Table S2 for full gene list and fold changes) using NPD and LPD stud male sperm. Online bioinformatics analysis revealed significant down regulation of 26 genes (Figure 6D; *P* < 0.05) associated with extracellular matrix (ECM) (Figure 6E), transcription factors (*Gli1, Twist1*) and growth factors (*Igf1, Pdgfa*) (Figure 6F), ECM protease function (Figure 6G) and bone ossification (Figure 6H) in LPD sperm.

## Discussion

We have investigated the association between a poor paternal diet at the time of conception and the bone development of his offspring. While several studies have found associations between paternal obesity,^26-28^ smoking^29^ and exercise^30^ with offspring growth, few studies have examined the impact on the bones themselves. Previously, we observed that a paternal LPD resulted in altered fetal bone density and hydroxyapatite crystal lattice parameters.^14^ In our current study, we extended these analyses to explore the specific sperm- and seminal plasma-mediated influences and whether changes in offspring bone morphology were maintained over two generations. We observed that from just 3 wk of age, neonatal offspring derived from LPD fed males displayed differential femur cortical and trabecular bone morphology as assessed using µ-CT. Furthermore, we observed sperm and seminal plasma-specific influences on bone hydroxyapatite crystal lattice parameters. In adulthood (16 wk of age), differences in femur bone morphology were still observed which associated with accompanying altered bone hydroxyapatite c-lattice parameters across the bone. Furthermore, in our second generation of offspring, differences in femur cortical and trabecular bone morphology were still evident. Finally, we analyzed paternal sperm mRNA content and seminal plasma composition to gain additional insight into the mechanisms linking paternal diet with offspring bone health. While we observed no difference in seminal plasma adiponectin, leptin or Tgfb1, we identified a down regulation in multiple ECM, transcription and growth factor and ossification genes in sperm from LPD males.

We observed no difference in F1 body weight or femur length between the groups at 3 wk of age. Furthermore, there was no significant effect of sex on body weight or bone dynamics analyzed. We have previously reported on litter size parameters in these mice, observing no difference in mean litter size or sex-ratio between groups.^16^ However, we did observe a reduction in femur volume in NL and LN offspring when compared with LL offspring. Furthermore, NL and LN offspring displayed differential measurements of trabecular thickness (Tb: Th), medullary cavity volume (Mv), degree of anisotropy (Da), and connectivity density (Conn.D) within the trabecular bone. The observation that offspring bone morphology was altered at just 3 wk of age suggests impairments in skeletal development during fetal or early neonatal life. Fetuses from dams fed a high-fat diet during pregnancy display signs of skeletal developmental delay, with decreased bone formation, volume and mineral density.^7,8^ In contrast, offspring of mice fed a maternal low-protein diet display enhanced volumes of high-density bone in late gestation.^9^ As this period represents a time of maximal bone growth and development, these changes may reflect altered profiles of osteoblast activity during this critical stage.^31^ However, in our study, all the dams and offspring were exposed to the same, standard laboratory chow. As such, the changes observed in our mice cannot be attributed to any dietary influences of the dam. Additionally, the mechanisms linking poor maternal and paternal diet to the programming of offspring bone health may be different. Interestingly, we recently reported changes in tissue lipid profiles from the same animals as assessed in the current study, with differences also evident from 3 wk of age.^19,32^ As such, fundamental changes in offspring metabolism, programmed in a sperm and seminal plasma-specific manner, may underlie the changes in neonatal bone health in our study. However, further studies are needed to connect fetal bone development and early offspring metabolic health in our model.

In addition to our morphometric analysis of F1 3 wk old offspring bones, we analyzed the hydroxyapatite lattice parameters of the same bones using SXRD. The mineral phase of bones in mammals derives from hydroxyapatite (HAp), Ca_10_(PO_4_)_6_(OH)_2._ During the early stages of bone development, formation and mineralization are critical for determining the quality of adult bones within an individual.^33^ We observed that NL and LN neonatal offspring displayed a reduced c-lattice parameter value when compared with NN neonates, as well as a reduced unit cell volume. Changes in c-lattice parameters can derive from ionic substitutions such as CO_3_^2−^, HPO_4_^−^, F^−^, SiO_4_^4−^, Mg^2+^, Na^+^.^34^ The replacement of the large phosphate (PO_4_^3−)^ ions by the smaller carbonate (CO_3_^2−)^ ions leads to changes in hydroxyapatite lattice structure resulting in an increased c-lattice parameter. As bone carbonate incorporation has been linked with increasing age,^35^ the reduction in c-lattice parameter seen in our NL and LN offspring might suggest these bones are less developed, which could have implications for bone health and strength in adulthood. However, additional studies would be needed to define the organization of any substitutions within our bones and their impact on bone structure and function. Interestingly, the significant reduction in femur c-lattice parameter observed in neonatal NL and LN bones were still evident in the adults. We also observed a more pronounced decrease in the c-lattice parameter in NL and LN bones as we scanned from the outer periosteum toward the medullary cavity. Multiple studies have identified lower hydroxyapatite lattice parameters in older bone.^36-38^ Similarly, studies of osteoporotic and non-osteoporotic bones showed a higher level of carbonate to phosphate ratio in samples of fractured osteoporotic bone when compared with non-osteoporotic samples.^39^ The stable c-lattice measurements observed across our NN bones might suggest a uniform hydroxyapatite structures. However, in the LL, NL, and LN samples, a higher degree of variability and increased shift in c-lattice might suggest a more variable hydroxyapatite composition, which could reflect an increased state of aging in these bones. Bone health can also be influenced by the individual’s metabolic status. Abdominal obesity, dyslipidaemia, hyperglycaemia, and hypertension are factors that have all been associated with changes in bone quality.^40^ In mice, dietary induced obesity results in a reduction in bone mineral density and content in the whole skeleton,^41^ coupled with alveolar bone loss, reduced osteoclastogenesis, and elevated inflammatory status.^42^ We have recently shown that the F1 adult mice analyzed in the current study become overweight, glucose intolerance, develop cardiovascular dysfunction and display perturbed tissue lipid profiles.^16,19,20,32^ It is therefore possible that these fundamental changes in metabolic homeostasis within our offspring influence their adult bone health.

We also observed significant differences in F1 body weight between males and females at 16 wk of age. However, only the NN offspring displayed a sexual dimorphism in femur bone length and volume. Further sexual-dimorphic differences were also observed in trabecular thickness, cortical bone volume and cortical bone cross-section thickness, however the extent of the sexual-dimorphism was not consistent between groups. Structural and morphometric differences between male and female bones are typically attributed to the action of sex and growth hormones, as well as mechanical loading.^43^ Significant differences between the sexes in response to fetal programming factors have been reported widely,^44^ with males typically more sensitive to environmental perturbations than females. However, additional transcriptomic, proteomic and/or biomechanical studies are required to define the extent to which paternal diet may differentially affect male and female bones.

A third major observation was that changes in bone morphology were still evident within our second generation offspring. LN offspring displayed an increased neonatal body weight, but a reduced femur volume and proportional length when compared with NN offspring at 3 wk of age. LN offspring, as well as NL and LL offspring also displayed altered trabecular and cortical bone morphology. Studies in rats have indicated that both paternal and maternal low birth weight programme offspring bone morphology within a first, but not second or third generation.^45^ We have shown that paternal LPD can programme offspring cardiovascular^20^ and metabolic^19,32^ health over two generations.

To explore the underlying mechanisms linking poor paternal diet at the time of conception with semen quality and offspring bone development further, we assessed seminal plasma composition and sperm mRNA content and in our stud males. Recent studies have identified significant relationships between the levels of specific adipokines and male reproductive function.^46^ In men, obesity has been associated with increased levels of several adipokines within the serum and seminal plasma and which correlated with functional seminal parameters.^47^ Furthermore, seminal molecules such as Tgfb1 has been shown to induce female reproductive tract immune tolerance and vascular remodeling responses, critical to enable post-implantation embryo development.^18^ Such changes in seminal plasma composition, coupled with changes in sperm mRNA content, could shape both the development of the embryo as well as the maternal uterine environment, impacting significantly on fetal growth and adult bone health.^48^ However, we observed no difference in seminal plasma concentrations of adiponectin, leptin, or Tgfb1 between our males. Expression analysis of a panel of 84 osteogenesis pathway genes within sperm from our stud males identified decreased expression of multiple genes associated with ECM status, transcription and growth factor and bone ossification. We observed decreased expression of several collagens (*Col1a1, Col1a2, Col3a1, Col4a1, Col5a1, Col14a1, Col15a1*). Collagens, particularly Type I collagen, are the primary protein component of bone’s organic matrix, providing the structural framework that allows bone to withstand stress and strain. Type 1 collagen also plays a role in regulating bone cell activity.^49^ Collagen also plays a vital role in maintaining bone health, and deficiencies or defects in collagen can lead to various bone diseases. Osteogenesis imperfecta, also known as brittle bone disease, is a genetic condition where collagen, particularly type I collagen, is either improperly formed or not produced in sufficient quantities, resulting in fragile bones that fracture easily. Additionally, we observed reduced expression of factors involved in ECM turnover such as *Ctsk, Bmp1, Mmp2*, and *Mmp9*. Mmp2 and Mmp9 have been shown to have a role in bone turnover and remodeling, with knock-out mice having shorter, weaker bones with reduced mineral content.^50,51^ Mmp2 and Mmp9 have also been shown to regulate the bioavailability and bioactivity of transforming growth factor β influencing bone hardness.^52^ Similarly, knock out cathepsin K (*Ctsk*) has been shown to result in mild osteopetrosis with increased trabecular and cortical bone mass due to impaired osteoclastic bone resorption.^53^ While our analyses identify potential sperm-mediated mechanisms, further studies are required to confirm the wider role of sperm transcript, ncRNA, and epigenetic status on offspring bone development and health.

## Conclusions

Our study provides novel insight into the impact of paternal nutrition on the bone development and health of his offspring. Our current study shows that both the sperm, and the seminal plasma, can program differential profiles of bone morphology. Importantly, our study shows that these effects are evident from just 3 wk of age, persist into adulthood and are maintained into a second generation. Interestingly, as for our analyses of cardio-metabolic health in these same mice,^16,19,20^ we observed more pronounced effects on offspring bones when the diet of the sperm and seminal plasma donors are mismatched (LN and NL). We hypothesize that if the priming of the uterus (by the seminal plasma) differs from that for the embryo (by the sperm) then the resultant mismatch induces a range of adaptive responses in the offspring, perturbing fetal growth and adult wellbeing. However, further detailed analyses of how poor paternal diet affects offspring bone health are needed.

## Supplementary Material

zqaf051_Supplemental_Files

## Data Availability

All data and materials used in the analysis are available in some form to any researcher for purposes of reproducing or extending the analysis.
