# Supplementary material for: Intergenerational Impact of Paternal Low-Protein Diet on Offspring Bone Health in Mice
Source: Function (Oxf). 2025 Oct 29;6(6):zqaf051. doi: 10.1093/function/zqaf051 (PMC12605816; doi:10.1093/function/zqaf051)
Supplement: zqaf051_Supplemental_Files [file zqaf051_supplemental_files.zip › Supplemental Table 1.docx]

**Supplementary data**

**Table S1: Ingredients and nutritional information of diets fed to male mice**

|  | **CD** | **LPD** |
| --- | --- | --- |
| **Energy density (kcal/g)** | 3.10 | 3.10 |
| **Proportional energy content (%)** |  |  |
| Protein | 16 | 8.4 |
| Fat | 23.4 | 23.1 |
| Carbohydrate | 60.5 | 68.5 |
| *of which sugars* | *20.7* | *23.6* |
| **Protein (% g/g)** |  |  |
| Casein | 18.0 | 9.0 |
| **Fats (% g/g)** |  |  |
| Corn Oil | 10 | 10 |
| **Carbohydrates (% g/g)** |  |  |
| Sucrose | 21.3 | 24.3 |
| Starch Maize | 42.5 | 48.5 |
| Cellulose | 5 | 5 |
| **Micronutrients (% g/g)** |  |  |
| Choline chloride | 0.2 | 0.2 |
| D,L-Methionine | 0.50 | 0.50 |
| Mineral mix (AIN-76) | 2 | 2 |
| Vitamin mix (AIN-76) | 0.5 | 0.5 |
| *Folic Acid | 1x10^-4^ | 1x10^-4^ |
| *Vitamin B12 | 5x10^-7^ | 5x10^-7^ |

Components contained within commercially available Vitamin mix AIN-76 that were supplemented in methyl-donor diets.

^†^ WD is commercially manufactured by Special Diet Services, diet code 829100. All other diets are custom formulations from Special Diet Services.
