# Supplementary figures and images for: Intergenerational Impact of Paternal Low-Protein Diet on Offspring Bone Health in Mice
Source: Function (Oxf). 2025 Oct 29;6(6):zqaf051. doi: 10.1093/function/zqaf051 (PMC12605816; doi:10.1093/function/zqaf051)

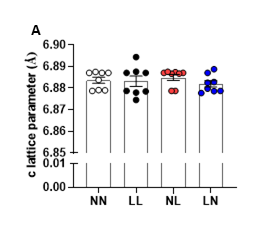

Supplement: zqaf051_Supplemental_Files [file zqaf051_supplemental_files.zip › Supplemental Fig 1.tif]
